# Eukaryotic Release Factor 3 Is Required for Multiple Turnovers of Peptide Release Catalysis by Eukaryotic Release Factor 1*

**DOI:** 10.1074/jbc.M113.487090

**Published:** 2013-08-20

**Authors:** Daniel E. Eyler, Karen A. Wehner, Rachel Green

**Affiliations:** From the Howard Hughes Medical Institute and the Department of Molecular Biology and Genetics, Johns Hopkins University School of Medicine, Baltimore, Maryland 21205

**Keywords:** Protein Synthesis, Ribosomes, Translation, Translation Control, Translation Release Factors, Translation, Translation Release Factors, Ribosomes, Protein Synthesis, Translational Control

## Abstract

Eukaryotic peptide release factor 3 (eRF3) is a conserved, essential gene in eukaryotes implicated in translation termination. We have systematically measured the contribution of eRF3 to the rates of peptide release with both saturating and limiting levels of eukaryotic release factor 1 (eRF1). Although eRF3 modestly stimulates the absolute rate of peptide release (∼5-fold), it strongly increases the rate of peptide release when eRF1 is limiting (>20-fold). This effect was generalizable across all stop codons and in a variety of contexts. Further investigation revealed that eRF1 remains associated with ribosomal complexes after peptide release and subunit dissociation and that eRF3 promotes the dissociation of eRF1 from these post-termination complexes. These data are consistent with models where eRF3 principally affects binding interactions between eRF1 and the ribosome, either prior to or subsequent to peptide release. A role for eRF3 as an escort for eRF1 into its fully accommodated state is easily reconciled with its close sequence similarity to the translational GTPase EFTu.

## Teaser

**Background:** eRF3 is an essential, conserved gene, whose essential function has remained obscure.

**Results:** eRF3 increases multiple turnover peptide release rates beyond the level expected from its stimulation of single turnover *k*_rel_.

**Conclusion:** eRF3 increases the efficiency of eRF1-mediated peptide release at limiting concentrations of eRF1.

**Significance:** This work contributes to our understanding of the essential *in vivo* role of eRF3.

## Introduction

Termination of protein synthesis in eukaryotes is mediated by three factors, eukaryotic release factors 1 and 3 (eRF1 and eRF3)^3^, and Rli1/ABCE1, which carry out the core activity of peptide release and connect peptide release with subsequent “recycling” of the ribosomal subunit (1, 2). The first factor, eRF1, connects the genetic code to translational output. Similar to a tRNA, eRF1 binds to the A site and reads the A site codon; after deciphering the stop codon, eRF1 triggers peptide release in a process dependent on its GGQ motif in domain 2 to catalyze the chemical hydrolysis reaction (3, 4). Although eRF1 is sufficient to promote peptide release *in vitro* (5–7), the *k*_cat_ for eRF1-only peptide release is slow relative to estimated rates of *in vivo* translation (0.014 s^−1^ *versus* 5–10 codons per second) (8, 9). Two accessory factors are known to increase the rate of peptide release *in vitro*. The first of these is the class 2 release factor, eRF3, which both accelerates the rate of peptide release and renders the process dependent on GTP hydrolysis (6, 7, 10). The second accessory factor is the ATPase Rli1/ABCE1, which accelerates the rate of peptide release in an ATP hydrolysis-independent fashion (1). This latter factor is then subsequently required for subunit splitting in an ATP hydrolysis-dependent reaction (1, 2). Because these two factors, eRF3 and Rli1 (RNase L-like 1), bind at overlapping sites on the ribosome, they are likely to promote distinct molecular steps in the termination and recycling processes (11–13).

Although eRF3 is essential and has been implicated in termination using a variety of approaches, the molecular role for eRF3 and its associated GTPase activity have been difficult to define (10). These difficulties in part stem from the fact that bacteria and eukaryotes both possess so called “class 2” GTPase release factors (RF3 and eRF3, respectively) that seem to share little in terms of origins or function. RF3 is a non-essential gene in bacteria that derives from the elongation factor G family of GTPases, whereas eRF3 is an essential gene in eukaryotes derived from the EFTu family of GTPases (14). Although both RF3 and eRF3 are GTPases, they belong to different ancestral families and have evolved independently (14). In a well defined *in vitro* system, RF3 has no effect on the *k*_cat_ for peptide release in bacteria but appears to modestly stimulate the removal of the class 1 release factor (RF1 or RF2) from the ribosome following peptide release (and prior to the recycling reaction) (15). Additionally, RF3 appears to play a key role in promoting a post-peptidyl transfer quality control step by directly accelerating the *k*_cat_ for peptide release (by as much as 50-fold) on ribosome complexes carrying a recent error in protein synthesis (16, 17).

eRF3 has also been characterized using *in vitro*-reconstituted translation systems and appears to stimulate peptide release in a GTP-dependent reaction (10, 18). However, how this GTP-dependent contribution of eRF3 is coordinated with the subsequent and interconnected recycling process in eukaryotes remains poorly understood. In addition, as factors involved in eukaryotic recycling have only recently been defined (1, 2, 19–21), it has not previously been possible to think about the likely integration of the steps of termination and recycling.

To better understand the molecular role of eRF3 in translation termination, we used an *in vitro*-reconstituted yeast translation system to determine the contribution of eRF3 to peptide release at both saturating and limiting concentrations of eRF1. At saturating levels of eRF1 (7), eRF3:GTP stimulates peptide release by ∼5-fold; both the absolute rates of peptide release and the stimulation afforded by eRF3 were consistent across all tested codons and codon contexts. More interestingly, we found that eRF3 accelerates the rate of peptide release >20-fold in assays where eRF1 is supplied at concentrations sub-stoichiometric to the ribosome. We also found that although eRF3 did not promote subunit dissociation in single turnover reactions, we noted that eRF1 remains physically associated with ribosomes and ribosomal subunits after termination when eRF3 is not present. These data are consistent with a role for eRF3 in broadly mediating binding interactions of eRF1 with the ribosome (by affecting either on or off rates or both); in particular, our data are consistent with a role for eRF3 in escorting eRF1 into its fully accommodated position in the A site of stop codon-programmed ribosomes. Such a molecular role for eRF3 would be consistent with the role played by EFTu in loading tRNAs onto the ribosome during elongation. Importantly, such a role would be distinct from the role played by RF3 in bacterial termination and post-peptidyl transfer quality control.

## EXPERIMENTAL PROCEDURES

### 

#### 

##### Proteins, tRNAs, and Ribosomes

The *SUP45* ORF (eRF1), without a stop codon, was PCR cloned into the NdeI and SmaI sites of the pTYB2 vector (New England Biolabs) and transformed into BL21(DE3) RIPL cells (Stratagene). Overnight cultures were diluted 1:200 and grown at 37 °C to an *A*_600_ of 0.6 and then chilled to 16 °C, and expression was induced overnight with 0.1 mm isopropyl 1-thio-β-d-galactopyranoside. Cells were harvested by centrifugation and resuspended in lysis buffer (20 mm HEPES-KOH, pH 7.4, 500 mm NaCl, 1 mm EDTA) and then either stored at −80 °C or lysed on a French press. The lysate was clarified at 9,000 × *g* for 5 min and at 30,000 × *g* for 30 min, and the clarified supernatant applied to a pre-equilibrated chitin resin (New England Biolabs). The resin was washed with 20 volumes of wash buffer (lysis buffer but with 1 m NaCl), and eRF1 was eluted overnight in 20 mm HEPES-KOH, pH 7.4, 500 mm NaCl, 1 mm EDTA, 50 mm DTT. The eluate buffer was exchanged on a HiTrap desalting column (GE Healthcare) into 20 mm HEPES-KOH, pH 7.4, 30 mm NaCl, 2 mm DTT, and applied to a MonoQ 5/50 GL column (GE Healthcare). After washing, bound protein was eluted with a linear gradient to 1 m NaCl in the same buffer. The major peak was full-length eRF1 and was subsequently applied to a Sephacryl S-100 HR 26/60 column (GE Healthcare) and eluted in 20 mm HEPES-KOH, pH 7.4, 100 mm potassium acetate, pH 7.5, 2 mm DTT, 10% glycerol. Purified protein was quantitated by absorbance at 280 nm and stored in aliquots at −80 °C.

A portion of the *SUP35* ORF (eRF3), from amino acids 166 through 685, was cloned into the NdeI and SmaI sites of the pTYB2 vector (New England Biolabs) and transformed into BL21(DE3) RIPL cells (Stratagene). Growth and induction were identical to the eRF1 purification, as described above. The purification strategy, including buffers, is as described for eRF1, up to the gel filtration step. A Sephacryl S-200 HR 26/60 column was used for the final step, and the buffer used is 20 mm HEPES-KOH, pH 7.4, 300 mm KCl, 5% glycerol, 0.1 mm EDTA, and 2 mm DTT. Purified protein was quantified by absorbance at 280 nm and stored in aliquots at −80 °C.

The methodology used for purification of ribosomes and other translation factors, model mRNAs, and charged tRNAs, was described in detail in Eyler and Green (7). The model mRNA used in this study used a small ORF with the sequence AUG UUC UNN N, where UNN N was the termination sequence indicated in the respective figures. Complexes were assembled and concentrated by pelleting through a sucrose cushion as described previously.

##### In Vitro Assays

Pre-steady state assays for peptide release were carried out in buffer E (20 mm Tris-Cl, pH 7.5, 100 mm KOAc, pH 7.5, 2.5 mm Mg(OAc)_2_, 0.25 mm spermidine, and 2 mm DTT) at 26 °C. In general, pretermination [^35^S]Met-Phe dipeptide complex was preincubated with 2 μm eRF3 and 1 mm GTP for 3 min prior to the addition of 1 μm eRF1. Aliquots were removed and quenched in 5% formic acid at the indicated time points. Reaction products were separated by electrophoretic TLC and quantitated on a phosphorimaging device. When monitoring subunit separation, complexes were prepared with ^32^P-labeled tRNA^Phe^ (22), and the reaction was followed using native gels (19). Multiple turnover assays were conducted in the same manner as single turnover reactions, except that eRF1 was added to a concentration of 2 nm, and the time course was longer. All reactions, except those specifically labeled as nucleotide-free, contained 1 mm guanine nucleotide.

The binding of stoichiometric eRF1 to termination complexes was analyzed as follows. Termination complexes were prepared and purified as described above and reacted for 20 min with eRF1. The complexes were then layered onto 5–20% sucrose gradients in reaction buffer. The gradients were centrifuged for 3 h at 40,000 rpm in an SW41 rotor (Beckman). Gradients were pumped and traces collected using an ISCO UA-6 apparatus. Fractions were collected and analyzed for the presence of eRF1 by Western blotting.

##### Production of Antibodies

Polyclonal antibodies to eRF1 and eRF3 were prepared from rabbit antisera produced by Covance. The antigens provided were purified *Saccharomyces cerevisiae* eRF1 and eRF3 produced in *Escherichia coli* as described above. The antibodies were purified via two affinity steps, the first being a protein A resin and the second being an eRF1 or eRF3 affinity resin. The affinity resins were prepared from activated Sepharose (GE Healthcare) and purified proteins. Manufacturer recommended protocols were followed for each resin.

##### Genetic Depletion of eRF3

A truncated version of eRF3 (*SUP35*) missing the first 253 amino acids was PCR amplified and placed under the control of the *GAL1*-inducible promoter in the pAG415GAL vector (*LEU2 CEN6 ARSH4*) (Addgene plasmid 14145, Susan Lindquist) resulting in the production of the conditional expression vector pAG415GAL-*erf3^ΔN253^*.

A *S. cerevisiae* heterologous disruption strain in which one allele of *SUP35* has been replaced with the *KAN* gene was obtained from Open Biosystems. One meiotic segregant (*SUP35*) of the genotype: BY474x *SUP35* (*lys2*Δ*0 leu2*Δ*0 his3*Δ*1 ura3*Δ*0*) and one meiotic segregant (*pGAL1::erf3^ΔN253^*) of the genotype: BY474x *sup35*Δ::*KAN* (*lys2*Δ*0 leu2*Δ*0 his3*Δ*1 ura3*Δ*0*)(pAG415GAL-*erf3^ΔN253^*) were generated for further use.

To genetically deplete the eRF3p^ΔN253^ protein, the depletion strain created above was grown in YPGR (1% yeast extract, 2% peptone, 2% galactose, 2% raffinose) at 30 °C, and the cells were then pelleted, washed two times with cold sterile MilliQ water, and diluted into YPD (1% yeast extract, 2% peptone, 2% glucose). The YPD cultures were then grown at 30 °C with continuous shaking.

##### Polysome Analysis

Cells were grown to mid log phase, harvested by vacuum filtration and flash frozen in liquid nitrogen. Lysis buffer (300 mm NaCl, 15 mm Tris-HCl, pH 7.5, 15 mm MgCl_2_, 1% Triton X-100, 0.1 mg/ml cycloheximide, 1 mg/ml heparin) was added to the samples while in liquid nitrogen. Frozen yeast pellets were ground using a freezer mill, and lysates were cleared at 8,400 × *g* at 4 °C for 5 min. Cleared lysates were layered onto 10–50% sucrose gradients (300 mm NaCl, 15 mm Tris-HCl, pH 7.5, 15 mm MgCl_2_, 0.1 mg/ml cycloheximide, 1 mg/ml heparin) with a 60% sucrose cushion. Gradients were spun in an SW-41 rotor at 40,000 rpm at 4 °C for 3 h and fractionated with an Isco FoxyR1 Retriever/UA-6 detector system. Protein was precipitated from each fraction by methanol precipitation.

##### Western Blot Analysis

Protein was separated on 10% SDS-PAGE gels and transferred to PVDF membranes. Standard ECL (GE Healthcare) Western blotting techniques were used. Western blot signals were detected on unflashed Hyperfilm ECL (Amersham Biosciences). Primary antibody and secondary antibody incubations were carried out in PBS buffer containing 5% milk and 0.1% Tween 20. Antibodies used in this study were as follows: rabbit anti-Erf1p (see above), rabbit anti-Erf3p (see above), goat anti-rpS6 (Santa Cruz Biotechnology), donkey anti-rabbit-HRP (Santa Cruz Biotechnology), and donkey anti-goat-HRP (Santa Cruz Biotechnology).

ImageJ analysis software was used to determine the relative levels of eRF1 and rpS6 detected by Western blot analysis. Images for quantitation were selected from multiple film exposures based on the criteria that the bands of interest display increased intensity upon increased exposure times. The intensity of the signal in each lane was quantitated three independent times and averaged together. Percent of total protein per fraction was calculated both as a percent of the total material loaded on the gradient and as a percent of the total material detected in all gradient fractions. Results from these two methods were close and revealed the same trends. The values displayed were determined by using the sum of all methods for detection.

## RESULTS

### 

#### 

##### The Rate Constant for Peptide Release (k_rel_) by eRF1 Is Modestly Stimulated by eRF3 and Is Dependent on GTP Hydrolysis

**FIGURE 1.**
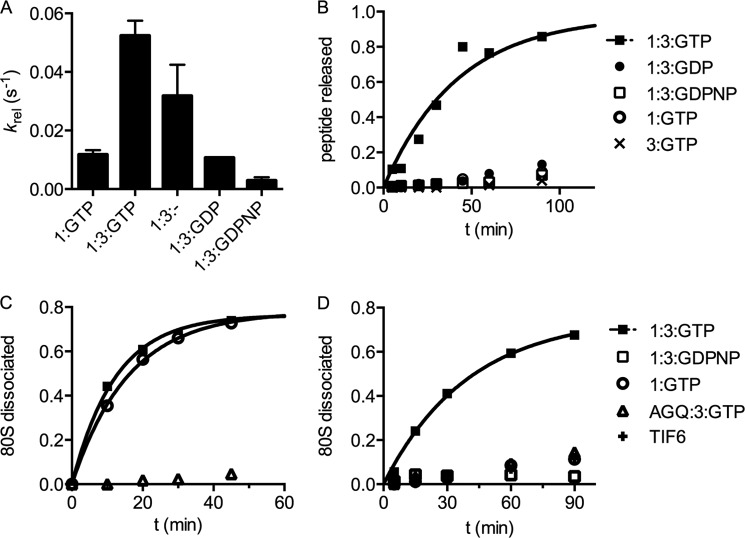
**eRF3 markedly stimulates the rate of multiple turnover peptide release by eRF1.** *A*, the rate constant for peptide release at saturating release factor concentrations depends on which factors and nucleotides are added. *1:GTP* indicates eRF1 (1 μm) and GTP, *1:3:GTP* indicates eRF1, eRF3 (2 μm), and 1 mm GTP, etc. *B*, multiple turnover peptide release depends on eRF3 and GTP. Limiting (2 nm) eRF1 was incubated with excess pre-termination complex (∼70 nm) and the fraction of dipeptide released was monitored as a function of time. eRF3 was added at saturating levels when indicated; nucleotides were at 1 mm. *C*, the rate of single turnover subunit dissociation does not depend on eRF3. Termination complexes were prepared with a ^32^P-labeled tRNA in the P site, and the fraction of subunits dissociated over time was monitored by native gel analysis. Factors were added at saturating concentrations as indicated in the legend. *D*, multiple turnover subunit dissociation depends on eRF3 and GTP. Termination complexes were prepared as in *C* and reacted with limiting eRF1, saturating eRF3, and nucleotides as in *B*.

We used a previously developed *in vitro*-reconstituted yeast translation system to evaluate the contributions of eRF3 to termination and recycling in eukaryotes (7). In this work, termination refers to peptide release, whereas recycling refers to steps that are necessary to split subunits and release the remaining components from the ribosome for subsequent rounds of initiation. As recycling necessarily involves multiple steps, we will carefully refer to these steps as subunit separation, and eRF1, mRNA or tRNA dissociation. As a first step, we used defined mRNAs to program yeast ribosome termination complexes (TCs) with a dipeptidyl tRNA (Met-Phe-tRNA^Phe^) in the P site and a UAA stop codon in the A site. When saturating levels of eRF1 (7) are added to these termination complexes, peptide release proceeds at a rate of 0.014 s^−1^ on the UAA stop codon (Fig. 1*A*, 1:GTP), and this rate is increased (5-fold) by the addition of saturating amounts of eRF3:GTP (Fig. 1*A*, 1:3:GTP).

We next addressed the question of the guanine nucleotide specificity of eRF3. Specifically, we asked whether GTP, GDPNP, and GDP promoted, permitted, or inhibited single turnover peptide release (*k*_rel_) by eRF1:eRF3. Although addition of eRF3:GTP increased *k*_rel_ by 5-fold over the eRF1-only rate, as mentioned above, the *k*_rel_ in the presence of eRF3:GDP is unchanged from the eRF1-only rate (Fig. 1*A*). Addition of GDPNP diminished *k*_rel_ below the eRF1-only rate (Fig. 1*A*), consistent with published data (6, 7, 18). We attempted to measure *k*_rel_ by eRF1:eRF3 in the absence of guanine nucleotides, expecting to observe the same rate as eRF1 alone and eRF1:eRF3:GDP. Curiously, the *k*_rel_ for this reaction was at an intermediate level of stimulation (3-fold) over the eRF1-only rate; however, the end point of this reaction (30%) was lower than expected (90%) (data not shown). These latter results are consistent with contamination of the apo-eRF3 sample by low levels of GTP that carry through the various purification steps. Although the nucleotide-free eRF3 (prepared through extensive dialysis in 10 mm EDTA) was determined to be >95% nucleotide-free by HPLC (data not shown), the pretermination complexes do not tolerate such stringent purification methods and thus cannot be eliminated as a source of contaminating GTP. Overall, our kinetic measurements are consistent with previously published qualitative results (6, 18) and indicate that GTP hydrolysis by eRF3 plays a modest role in determining the rate of peptide release at saturating concentrations (7) of the release factors.

##### eRF3 Makes Substantial Contributions to Multiple Turnover Peptide Release when eRF1 Is Sub-stoichiometric Relative to Termination Complexes

We next evaluated the effect of eRF3 in reactions where eRF1 was present at sub-stoichiometric concentrations (2 nm) relative to the termination complex (∼70 nm). Under these conditions, eRF1 must perform multiple rounds of peptide release (*i.e.* turnover) on different ribosome complexes for the reaction to proceed to completion. The effect of eRF3 under these conditions is dramatic; there is little release of the dipeptidyl-tRNA-programmed termination complexes in the absence of eRF3:GTP. We note that GTP was included in the eRF1-only reaction, allowing us to rule out the action of contaminating GTPases. Moreover, in this multiple turnover reaction, eRF3 with hydrolyzable GTP is required for stimulation of the release reaction by eRF3; eRF3:GDPNP and eRF3:GDP do not support multiple turnovers of eRF1 (Fig. 1*B*). The initial rate of the eRF1:eRF3:GTP reaction is at least 20-fold above the eRF1-only rate; this enhancement ratio is a lower limit because the eRF1-only rate is indistinguishable from the background rate.

##### eRF3 Uniformly Contributes to the Single Turnover Rates for Peptide Release (k_rel_) by eRF1 on Different Tetranucleotide Terminator Elements

**FIGURE 2.**
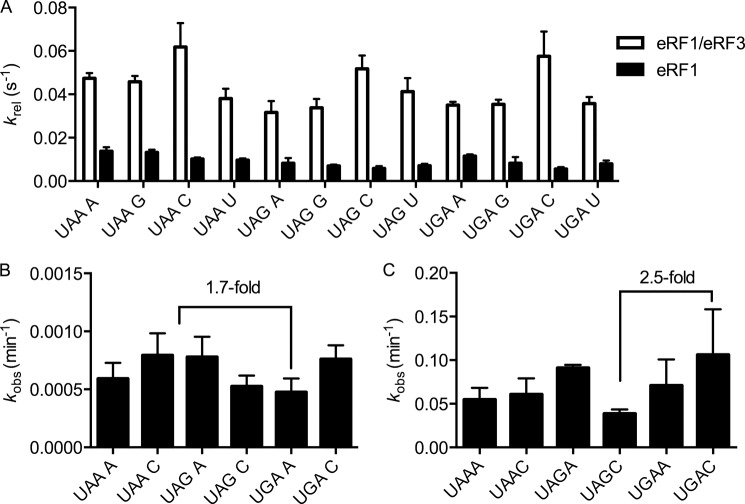
**Stop codon and the distal nucleotide at position +4 have small effects on the rate of peptide release.** *A*, the rate constant for peptide release at saturating release factor concentrations depends slightly on the stop codon and the nucleotide at position +4. The *white bars* indicate the rate of peptide release mediated by eRF1:eRF3:GTP, whereas the *black bars* indicate the rate of peptide release mediated by eRF1 alone. *B*, the observed rates of multiple turnover peptide release by eRF1 varies <2-fold across a subset of stop codons and +4 nucleotides. Reactions were carried out as in Fig. 1*B*. Observed rates are plotted; *error bars* represent the S.E. *C*, the observed rates of multiple turnover peptide release by eRF1 and eRF3 depend slightly on stop codon and the nucleotide at position +4. Reactions were carried out as described in Fig. 1*B*. Observed rates are plotted; *error bars* represent the range.

Previous studies had suggested that the sequence context of the stop codon differentially impacts recognition (*i.e.* binding) or catalysis by eRF1 and eRF1:eRF3 (23). Because our peptide release experiments had utilized only one stop codon in one sequence context, we asked whether the modest stimulation of peptide release by eRF3 could be generalized to other stop codons and contexts. For this analysis, we generated termination complexes containing each of the three stop codons (UAA, UAG, and UGA) in the A site followed by the four different nucleotides at position +4 (a total of 12 sequences). Rate constants for peptide release were then determined using both saturating eRF1 alone, as well as saturating eRF1 in combination with saturating levels of eRF3 and GTP (Fig. 2*A*). Rates of eRF1-only peptide release ranged from 0.006 s^−1^ on UAG C to 0.014 s^−1^ on UAA A, a range of 2.3-fold. Addition of eRF3 yielded increased overall rates ranging between 0.03 s^−1^ on UAG A and 0.06 s^−1^ on UAA C, a range of 2-fold. The stimulation afforded by eRF3 was between 3- and 10-fold on UGA A and UGA C, respectively, whereas the average stimulation by eRF3 was 5-fold. These modest effects of eRF3 on codon recognition in the *in vitro* system do not correlate particularly well with the earlier *in vivo* studies (23).

Although stop codon sequence and context did not dramatically affect peptide release rates in the single turnover assay, it remained possible that they could have an effect on the multiple turnover release reaction. This possibility was addressed by evaluating peptide release rates at limiting concentrations of eRF1, as described above, on all three stop codons, with either an A or a C at position +4. Consistent with the modest effects of codon and context in the single turnover peptide release assay (Fig. 2*A*), the observed rates of peptide release did not vary >2.5-fold on the different mRNA templates under multiple turnover conditions where contributions of binding affinity should be critical (Fig. 2, *B* and *C*).

##### Subunit Dissociation Requires eRF1 but Not eRF3

Because eRF3 stimulates the rate of the multiple turnover reaction by at least 20-fold, and the increased rate of peptide release (*k*_rel_) contributed by eRF3 is only 5-fold, it seems likely that eRF3 contributes to another step independent of peptide release *per se* including the equilibrium binding (on or off rates) of eRF1 to the termination complex or subunit dissociation following peptide release (and the potentially correlated dissociation of eRF1).

To address these possibilities, we asked whether eRF1 on its own can promote subunit dissociation and whether eRF3 accelerates the rate of this particular step. For this, we used a subunit dissociation assay in which we follow subunit separation via native gel electrophoresis (19, 24). In a single turnover transient kinetic assay with saturating eRF1, we found that subunits spontaneously separate after peptide release by eRF1 and that eRF3 does not accelerate this step (Fig. 1*C*). Moreover, subunit dissociation is accelerated by peptide release, as the catalytically inactive AGQ variant of eRF1 (6) is significantly slower. This result argues that the eRF3-mediated stimulation of peptide release in the multiple turnover reaction is not the result of an effect on subunit dissociation.

This question was further explored by evaluating the rate of subunit dissociation at limiting concentrations of eRF1 (Fig. 1*D*). As in the multiple turnover peptide release experiment, limiting eRF1 was not able to perform multiple rounds of subunit dissociation; addition of eRF3 and GTP allowed subunit dissociation to proceed to completion. This reaction was not promoted in the presence of the non-hydrolyzable analog GDPNP or with the catalytically inactive eRF1 variant. These data suggest that subunit dissociation is not directly stimulated by eRF3 but rather that subunit dissociation is licensed by the completion of peptide release.

##### eRF3 Decreases the Amount of eRF1 Associated with Post-termination Ribosomes in Vitro and in Vivo

**FIGURE 3.**
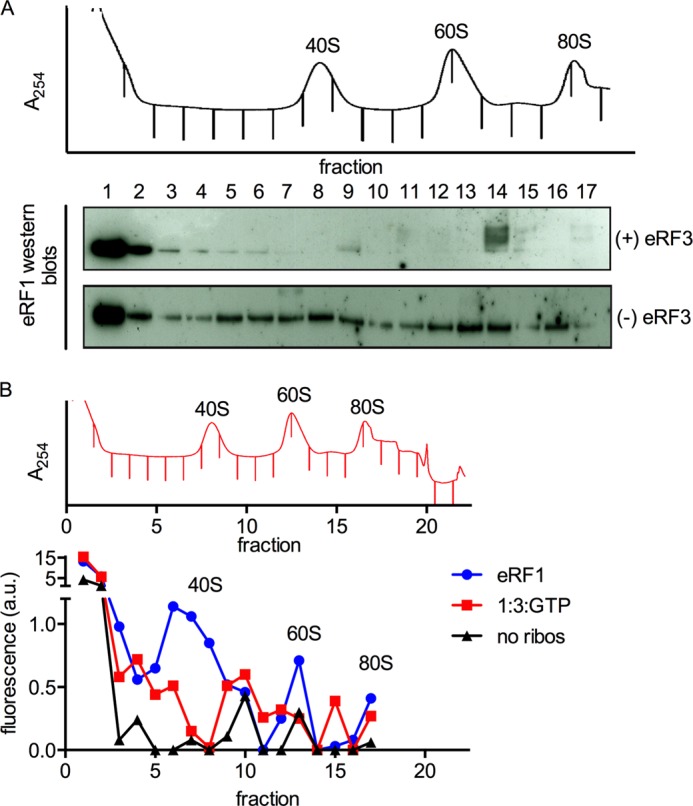
**eRF1 remains associated with ribosomal subunits after peptide release and subunit dissociation.** *A*, eRF1 is detected throughout the gradient in the absence of eRF3. Termination complexes (∼70 nm) were reacted with stoichiometric eRF1, with or without eRF3 (1 μm), and separated by sucrose density gradient centrifugation. A trace of absorbance at 254 nm is shown, and the positions of the ribosomal components are labeled. Fractions were collected as indicated by the *tick marks* in the absorbance trace and analyzed for the presence of eRF1 by Western blotting with standard ECL techniques with an antibody against eRF1. eRF1 is almost exclusively at the top of the gradient in the presence of eRF3 but co-sediments substantially with the 40S, 60S, and 80S peaks in the absence of eRF3. *B*, eRF1 is detected in the 40S and 60S peaks in the absence of eRF3. Termination complexes were prepared and reacted with eRF1 and/or eRF3, and sucrose density gradient centrifugation was performed as described in *A*. The results of a quantitative Western blot are shown below the 254 nm absorbance trace. “No ribos“ indicates eRF1 was centrifuged without termination complexes as a negative control. Western blotting was performed using fluorescently labeled secondary antibodies (LICOR). The signal was quantitated using Odyssey and plotted on the *y* axis in arbitrary units of fluorescence intensity. Note that the *y* axis is broken to show the fluorescence in fractions 1 and 2.

In light of models for RF3 function in bacterial translation, we asked whether the presence of eRF3 impacted the amount of eRF1 bound to ribosomes following their involvement in a termination reaction. To address this question, we prepared dipeptide pretermination complexes and reacted them with stoichiometric amounts of either eRF1:GTP or eRF1:eRF3:GTP and separated the products by sucrose density gradient centrifugation. We followed the position of ribosomal subunits through their absorbance at 254 nm (Fig. 3) and detected eRF1 in the fractions by Western blotting with a polyclonal antibody against eRF1. We used both standard chemiluminescence protocols (Fig. 3*A*) and fluorescently labeled secondary antibodies (Fig. 3*B*) and observed similar results. The majority of eRF1 is found in the light fractions at the top of the gradient, consistent with the results of others (6). Strikingly, in the presence of eRF3:GTP, little to no eRF1 is seen in the gradient associated with ribosome particles, whereas in its absence, eRF1 is seen throughout the gradient, particularly in heavier fractions containing 40S, 60S, and 80S particles (Fig. 3). We attribute the spreading of eRF1 throughout the gradient to eRF1 dissociation from post-TCs during centrifugation, which is consistent with the results of other groups (6). The percentage of eRF1 retained on ribosomes without eRF3 in this assay is low, due partly to the stringency of the assay and to the stoichiometric levels of eRF1 used to facilitate detection. Despite these limitations, the result clearly reflects the greater stability of eRF1-bound post-TCs in the absence of eRF3.

**FIGURE 4.**
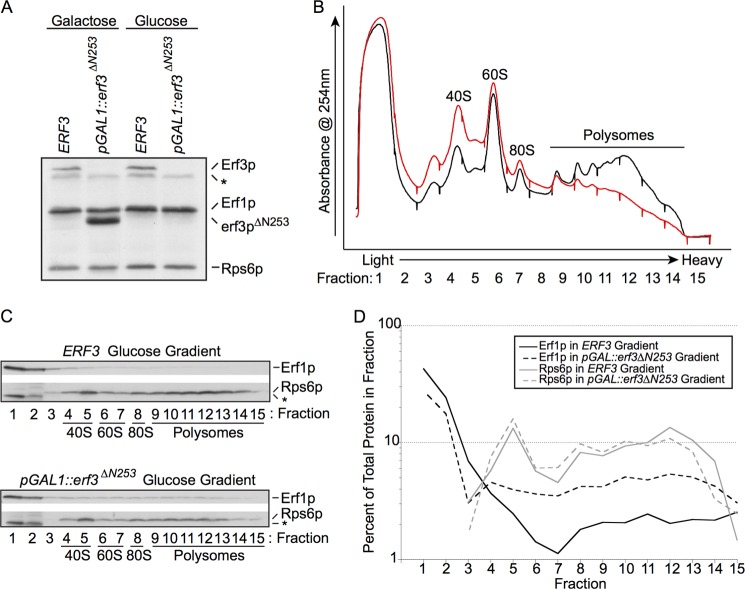
**Depletion of eRF3 *in vivo* results in redistribution of eRF1 on polysome gradients.** *A*, Western blot analysis of total cellular lysates prepared from wild type *ERF3* (the *SUP35* gene) and *pGAL1*::*erf3^ΔN253^* strains grown for 8 h in galactose (permissive for both) or glucose (erf3^ΔN253^ depletion). Equal *A*_260_ units of each lysate were loaded on the gel. The *asterisk* indicates a background band. *B*, cellular lysates prepared from wild type *ERF3* (*black line*) and *pGAL1*::*erf3^ΔN253^* (*red line*) strains grown for 8 h in glucose (erf3p^ΔN253^ depletion). Equal *A*_260_ units of each lysate were separated on sucrose density gradients as described under “Experimental Procedures.” Absorbance peaks (254 nm) that correspond to the ribosomal 40S and 60S subunits as well as 80S monosomes and polysomes are indicated. Fraction 1 is the top of the gradient, and fraction 15 is the bottom of the gradient. *C*, Western blot analysis of the sedimentation of eRF1 and Rps6p during sucrose gradient analysis of wild type *ERF3* and *pGAL1*::*erf3^ΔN253^* strains grown in glucose. The *asterisk* indicates a background band observed in fractions 1 and 2 prior to detection with the rpS6 antibody. *D*, quantitation of the percent of total protein found in each sucrose gradient fraction for the Western blots shown in *B*.

To investigate the impact of eRF3 on the association of eRF1 with post-termination ribosomal complexes *in vivo*, we constructed a yeast strain in which a variant of eRF3 is under the control of a galactose-inducible, glucose-repressible promoter. Growth of this strain in glucose for 8 h results in eRF3 levels being diminished below the limits of detection and a noticeable decrease in polyribosomes (Fig. 4, *A* and *B*). Consistent with our *in vitro* results (Fig. 3) and published *in vivo* results (25), we find that eRF1 from actively translating wild type yeast lysates strongly accumulates in the lightest fractions at the top of sucrose gradients but is also distributed in fractions containing ribosomal particles (Fig. 4*C*, *lanes 4–14*). Upon depletion of eRF3 from yeast cells, we find that eRF1 levels are somewhat increased in heavier fractions containing ribosomal particles (Fig. 4*C*, *lanes 4–15*). These results are quantitated in Fig. 4*D*. Taken together, our *in vitro* and *in vivo* results indicate that eRF3 decreases the association of eRF1 with ribosomal components following peptide release or subunit splitting either through an active (catalytic) or passive (trapping) mechanism.

## DISCUSSION

**FIGURE 5.**
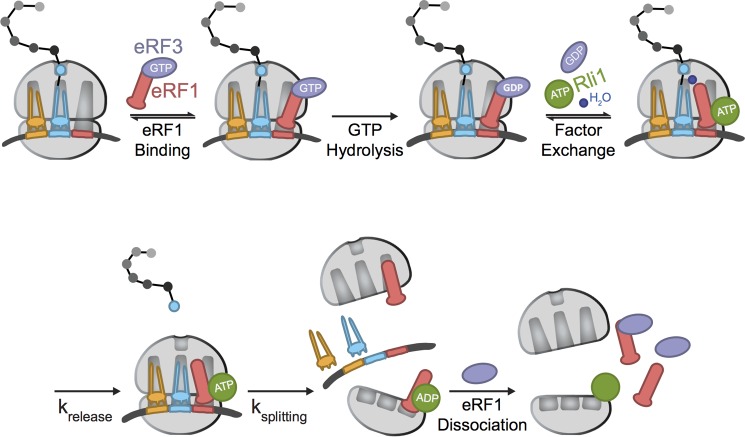
**Model of the roles of eRF1 and eRF3 in eukaryotic termination.** A simplified model for eukaryotic termination, emphasizing the steps at which eRF1 may be influenced by eRF3. *k*_release_ and *k*_splitting_ refer to the processes of peptide release and subunit dissociation, respectively. These two steps could be directly measured in our kinetic analysis; binding steps (indicated with equilibria) have not been directly monitored here.

Numerous hypotheses have been advanced regarding the essential function of eRF3 in eukaryotic cells (6, 15, 23, 26). To gain further insight into eRF3 function, we systematically characterized the contributions of eRF3 to the kinetics of eRF1-mediated peptide release and subunit dissociation. We have found that eRF3:GTP makes modest (5-fold) contributions to the rate constant for peptide release (*k*_rel_) catalyzed by eRF1 (Fig. 5, *k*_release_) and that this effect is broadly consistent across stop codons and different contexts. When eRF1 is limiting, however, the effect of eRF3:GTP is more substantial, with >20-fold increases in the initial rates (*k*_obs_) of the reaction and substantial increases in end point from <10% to 90%.

These data can be rationalized by several different potential roles for eRF3 in the termination and recycling events during translation. First, it is possible that eRF3:GTP is important for promoting efficient binding and accommodation of eRF1 to the ribosome termination complexes (Fig. 5, “eRF1 binding” through “factor exchange”). Such a role for eRF3 would be most similar to that of EFTu, its close homolog, which chaperones aminoacyl tRNAs into the A site during each step in the elongation cycle (14, 27). Another possibility is that eRF3 promotes some post-termination step, including either the actual subunit dissociation step or the dissociation of eRF1 from the large and small subunits following subunit dissociation (Fig. 5, *k*_splitting_ and eRF1 dissociation). These roles would be more similar to that proposed for the bacterial termination factor GTPase RF3, which appears to increase the dissociation of RF1/RF2 from post-termination ribosome complexes (15). These options were experimentally addressed where possible and are discussed below.

To address whether eRF3:GTP increases the actual binding of eRF1 to the ribosome requires a quantitative binding assay that we do not have currently. However, it is generally thought that eRF3 is bound to eRF1 in the cell (10, 28) and that this complex binds to ribosome termination complexes. As we discussed above, peptide release is specifically promoted on complexes with a termination codon in the A site, and eRF3 contributes modestly to the rate for this reaction. The fact that the multiple turnover reaction is more substantially stimulated by eRF3:GTP is certainly consistent with a model where eRF3:GTP promotes a step prior to peptide release such as binding or accommodation. Interestingly, earlier studies found there to be no significant differences in the *K*½ for eRF1 interacting with termination complexes in the presence or absence of eRF3 (7). Given the 5-fold stimulation of *k*_rel_ in the presence of eRF3, it is likely that eRF3 alters the apparent *k*_on_ (an increase) or *k*_off_ (a decrease) of eRF1 to the ribosome. We emphasize that these are apparent on- and off-rates that reflect the contributions of all steps from initial binding up to, but not including peptide release. Thus, eRF3:GTP could increase any of the forward rates, or decrease any of the reverse rates, to increase the apparent affinity of eRF1 for the ribosome. Although not a direct binding assay *per se*, this argument is consistent with an EFTu-like role for eRF3 in termination, where GTP hydrolysis in this case allows for “accommodation” of the eRF1 into its binding site. Such an interpretation would also be consistent with recent cryo-EM studies showing eRF1:3:GDPNP trapped in a pre-accommodation state on the ribosome during delivery into the stop codon-programmed A site.^4^ Moreover, our recent studies suggested that Hbs1, a translational GTPase involved in No-Go-Decay, also functions similarly to EFTu, in this case escorting Dom34 into the A site of relevant cellular targets (1, 11, 19). In the absence of more detailed quantitative assays that can distinguish the multiple steps preceding peptide release, we cannot provide exclusive support for such a model.

We next asked whether eRF3:GTP affected events subsequent to the actual termination event. Our earlier work had argued for a model where eRF3 departs after GTP hydrolysis and is replaced by the ATPase Rli1, which binds to an overlapping site (Fig. 5, factor exchange). Peptide release is accelerated by Rli1:ATP and then subunit dissociation is driven by ATP hydrolysis (with the help of still bound eRF1) (Fig. 5, *k*_release_ and *k*_splitting_) (1, 2). However, given that we find here that eRF3:GTP directly stimulates the rate constant for multiple turnover peptide release, it seemed possible that eRF3 might, under certain conditions, promote subunit dissociation in addition to peptide release. When this potential role was explored in our *in vitro*-reconstituted subunit dissociation assay, we saw no evidence to support this model; single turnover subunit dissociation occurs at the same rate in the presence of eRF1 alone or with eRF3:GTP present.

The final possibility that we tested was whether eRF3:GTP helps in promoting the dissociation of eRF1 from the ribosomal subunits following the subunit dissociation reaction (Fig. 5, eRF1 dissociation). In this potential role, eRF3 would increase the effective concentration of eRF1 available to perform subsequent rounds of peptide release by trapping eRF1 in a productive complex as it dissociates from post-termination ribosomal particles. These ideas were supported by both *in vitro* and *in vivo* experiments (Figs. 3 and 4). First, using the *in vitro*-reconstituted translation system, we asked whether eRF1 tends to partition with either large or small subunits following the peptide release and subunit dissociation reactions. Without eRF3, eRF1 was clearly associated with both the large and small ribosomal subunits and even with the few remaining 80S particles; addition of eRF3:GTP very effectively reduced the amount of eRF1 co-sedimenting with the 40S, 60S, and 80S peaks under the chosen *in vitro* conditions. Similarly, when eRF3 is depleted in yeast cells using a glucose-regulated promoter, we see that eRF1 tends to accumulate more in the subunit, monosome, and polysome fractions.

Collectively, our data are consistent with two models for eRF3 function. The first model is that eRF3 facilitates the dissociation of eRF1 from post-TCs. The second model is that eRF3 accelerates the association of eRF1 with pre-TCs. It must also be noted that these two models are not mutually exclusive. In the first model, eRF3:GTP promotes the multiple turnover reaction by releasing sequestered eRF1 from ribosomal particles following the subunit dissociation reaction. Although this might occur via an active, catalytic “eRF1 removal” function of eRF3 as proposed previously (15), a more likely alternative model to explain both the *in vivo* and *in vitro* sucrose gradient analyses is that eRF3 has an affinity for eRF1 that is sufficient to trap eRF1 as it naturally dissociates from the ribosome population. We further note that the fact that eRF1 seems to partition equally well with 40S and 60S subunits, whereas the known GTPase activating center on the ribosome is found only on the large (60S) ribosomal subunit, makes us cautious in over-interpreting these eRF1 partitioning experiments as an explanation for the increases in the rates of the multiple turnover release reactions.

Indeed, it seems more likely that the requirement for GTP is at the eRF1 ribosome association stage (Fig. 5, eRF1 binding through factor exchange) and that promotion of the multiple turnover reaction by eRF3:GTP derives from increases in the apparent affinity of the interaction with eRF1 conferred by the class 2 release factor. These ideas will eventually be tested with in robust assays that measure the equilibria between different eRF1 states on the ribosome, in the presence and absence of eRF3.

eRF3 is an essential translational GTPase that functions in the final events of protein synthesis. Our data provide strong biochemical evidence that eRF3 is essential in promoting a multiple turnover peptide release reaction in a fashion that depends on GTP hydrolysis. Although we provide data to support the idea that eRF3 is important in helping to dissociate eRF1 from ribosomal particles following subunit splitting (Fig. 5, *k*_splitting_), we suspect that eRF3 more significantly promotes effective binding of eRF1 to the ribosome before peptide release, an idea that is well supported by the *k*_rel_ and *K*½ values determined for the reaction. Indeed, the homology between eRF3 and EFTu provides a compelling argument for eRF3:GTP contributing to the multiple turnover termination reaction by increasing the binding affinity of eRF1 to the point where it effectively engages ribosomal termination complexes (Fig. 5, eRF1 binding). Further strengthening this viewpoint are data indicating that eRF1 substantially changes conformation when complexed with eRF3; these changes may very well promote ribosome binding (29). In moving forward, these data help to define the roles of eRF3 in termination and to explain its essential functions *in vivo*.

## Footnotes

4 R. Beckmann and R. Green, personal communication.

## Footnotes

3 The abbreviations used are:

eRF3: eukaryotic release factor 3
EFTu: elongation factor Tu
*k*_rel_: the rate constant for single turnover peptide release
*k*_obs_: the observed rate of reaction
TC: termination complex.
